# Guillain-​Barré Syndrome Associated with COVID-19 Vaccination

**DOI:** 10.3201/eid2712.211634

**Published:** 2021-12

**Authors:** Shih-Chieh Shao, Chien-Ho Wang, Kai-Cheng Chang, Ming-Jui Hung, Hui-Yu Chen, Shu-Chen Liao

**Affiliations:** Keelung Chang Gung Memorial Hospital, Keelung, Taiwan (S.-C. Shao, C.-H. Wang, M.-J. Hung, S.-C. Liao);; National Cheng Kung University College of Medicine, Tainan, Taiwan (S.-C. Shao, K-C, Chang);; Linkou Chang Gung Memorial Hospital, Taoyuan, Taiwan (K.-C. Chang, H.-Y. Chen);; Chang Gung University College of Medicine, Taoyuan (M.-J. Hung, S.-C. Liao)

**Keywords:** COVID-19, coronavirus disease, SARS-CoV-2, severe acute respiratory syndrome coronavirus 2, viruses, respiratory infections, zoonoses, Taiwan, vaccine-preventable diseases, GBS, Guillain-​Barré syndrome, vaccines, vaccination

## Abstract

We conducted a multi-institutional study in Taiwan and a systematic review of the literature for reports of Guillain-​Barré syndrome after coronavirus disease vaccination. This condition, mostly the classic form and the acute inflammatory demyelinating polyneuropathy subtype, has been reported in 39 cases and has occurred within 2 weeks of vaccine administration.

Guillain-Barré syndrome (GBS), an immune-mediated polyradiculoneuropathy with a ≈5% mortality rate, has an incidence worldwide of 0.81–1.91 cases/100,000 person-years (*1*). GBS has been reported to be associated with coronavirus disease (COVID-19) vaccination, but a comprehensive summary regarding this rare adverse event is still lacking. To determine clinical features of GBS associated with COVID-19 vaccination, we conducted hospital-based investigations in Taiwan along with a systematic review of published case reports.

We analyzed electronic medical records data from Taiwan’s largest multi-institutional healthcare system, including 9 branches of Chang Gung Memorial Hospital (*2*), where healthcare workers received first-priority COVID-19 ChAdOx1-S vaccine (Oxford/AstraZeneca, https://www.astrazeneca.com) starting March 22, 2021. We included healthcare workers vaccinated during March 22–May 31 and followed them for 30 days after vaccination. We identified GBS cases on the basis of code G610 from the International Classification of Disease, 10th Revision, Clinical Modification, or spontaneous adverse drug reaction reporting systems within the hospitals. Two authors (C.H.W. and S.C.L.) confirmed diagnosis and classification of GBS cases through chart reviews (*3*,*4*). This study was approved by the Institutional Review Board of Chang Gung Medical Foundation (approval no. 202101087B0).

To summarize clinical features of published cases from literature, we searched PubMed and Embase for reports posted through August 17, 2021, using relevant key terms such as “COVID-19,” “Guillain-​Barré syndrome,” and “vaccine” with suitable MeSH terms. Two independent reviewers (S.C.S., C.H.W.) performed the study selection and data extraction; a third-reviewer (S.C.L.) settled any differences between them. We excluded cases with coexisting COVID-19 or preexisting GBS. We included only publications with reports of clinical features related to GBS. We described basic characteristics, laboratory data, pathologic reports, treatment patterns, and prognosis of GBS cases associated with COVID-19 vaccination. The study protocol of this systematic review is published on PROSPERO (https://www.crd.york.ac.uk/PROSPERO/display_record.php?RecordID=265479).

We included 18,269 healthcare workers (mean age 40.6 years, range 18–87 years; 67.5% were women) who received ChAdOx1-S vaccine during the study period. After these 18,257 first-dose and 544 second-dose vaccinations, we identified 1 GBS case after a first dose of ChAdOx1-S vaccine in 1 of the hospitals participating in the study.

After a systematic review of published literature (Figure), we included 17 publications reporting an additional 38 cases of GBS related to COVID-19 vaccination (India, 10 cases; United Kingdom, 11 cases; Mexico, 7 cases; United States, 3 cases; France, 1 case; Italy, 3 cases; Malta, 1 case; Turkey, 1 case; and Qatar, 1 case) (Appendix Table). Including the case in Taiwan, these 39 cases occurred in persons with a mean age of 57.8 (range 20–86) years; 56.4% were male. Most of the reported case-patients received ChAdOx1-S (25/39), followed by BNT162b2 (12/39) (Pfizer-BioNTech, https://www.pfizer.com), Ad26.COV2.S (1/39) (Johnson & Johnson, https://www.jnj.com), and CoronaVac (1/39) (Sinovac Biotech, http://www.sinovac.com). The GBS rate after COVID-19 vaccination ranged from 1.8 to 53.2 cases/1 million doses. The initial symptoms of GBS included myalgia (12/39), paraparesis (5/39), quadriparesis (22/39), paresthesia (28/39), and facial palsy (23/39), and symptoms of dysautonomia also were observed during hospitalizations (3/39). The average time from vaccination to symptom onset was 11.3 days. A total of 34 case-patients received lumbar puncture; 30 had manifestations of albuminocytologic dissociation in the cerebrospinal fluid.

**Figure:**
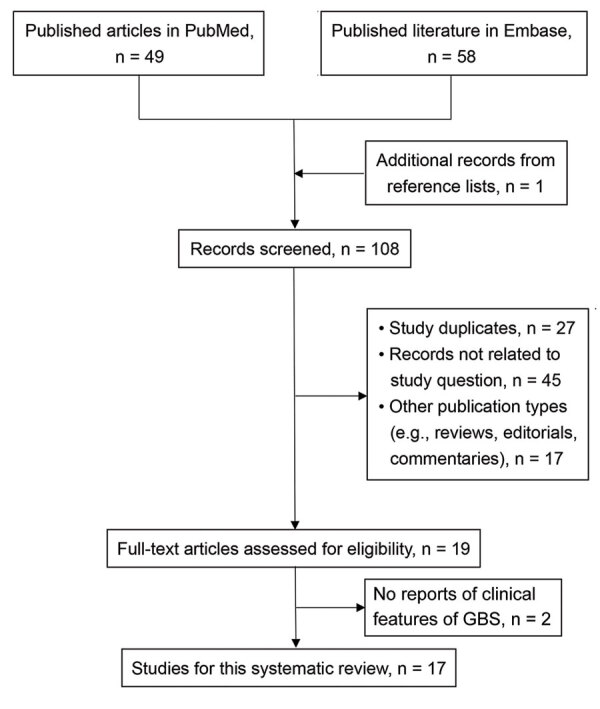
Systematic review of published literature in study of Guillain-​Barré syndrome associated with coronavirus vaccination, 2021. GBS, Guillain-​Barré syndrome.

On the basis of the clinical diagnostic classification of GBS, we found that most case-patients had the classic form (22/39), followed by bilateral facial palsy with paresthesia (12/39), the paraparetic form (4/39), and GBS–Miller Fisher syndrome overlap variant (1/39). We defined all classic and paraparetic forms of GBS (26/26) as level 1 or 2 on the basis of the Brighton criteria (*5*). We identified the GBS subtype in 33/39 cases by electrophysiological examination; most reported case-patients had a diagnosis of acute inflammatory demyelinating polyneuropathy (23/33), followed by acute motor and sensory axonal neuropathy (4/33) and acute motor axonal neuropathy (3/33). For GBS management, 33 case-patients received intravenous immunoglobulin and 2 received plasmapheresis. One case-patient died; 9 case-patients required mechanical ventilation during hospitalization. The scores on the GBS disability scale (*5*) were only available for 30 cases; 12 scored >4 (i.e., indicating bedridden or chair-bound status) during follow-up or after discharge.

Similar to previous reviews on GBS associated with COVID-19, we found that both COVID-19 and COVID-19 vaccination mostly cause the classic form of GBS (under the clinical diagnosis classification) and the acute inflammatory demyelinating polyneuropathy subtype (based on electrodiagnostic features) within 2 weeks of infection or vaccination (*6*–*8*). However, the bilateral facial palsy with paresthesia variant and initial onset symptoms of facial diplegia were more frequently found in GBS case-patients after COVID-19 vaccination.

Case series and reports can indicate safety issues and outline clinical features of diseases, but they cannot establish robust causal relationships between COVID-19 vaccination and GBS. Despite the benefits (e.g., increase in the number of persons not susceptible to infection and decrease in severe outcomes after infection) of COVID-19 vaccination far outweighing the potentially severe adverse events after infection (*9*), our findings highlight the need for vigilance in patients with neurologic symptoms after COVID-19 vaccination and for postvaccination surveillance programs to assess causality of GBS.

Appendix Additional information about Guillain-​Barré syndrome associated with COVID-19 vaccination.
