# Supplementary material for: Guillain-​Barré Syndrome Associated with COVID-19 Vaccination
Source: Emerg Infect Dis. 2021 Dec;27(12):3175–8. doi: 10.3201/eid2712.211634 (PMC8632191; doi:10.3201/eid2712.211634)
Supplement: Appendix — Additional information about Guillain-​Barré syndrome associated with COVID-19 vaccination. [file 21-1634-Techapp-s1.pdf]

Article DOI: https://doi.org/10.3201/eid2712.211634

Guillain-Barré Syndrome Associated with COVID-19 Vaccination

Appendix

Appendix Table. Clinical features of GBS associated with COVID-19 vaccination*

Author name (country)	GBS rate	Age, y	Sex	Vaccine	Vaccination to symptom onset, d	Initial symptoms (dysautonomia)	Albuminocytologic dissociation (CSF protein, mg/dl)	Clinical classification	Electro-diagnosis	Brighton criteria†	Antiganglioside antibody	Treatment (drug dose)	Mechanical ventilation	Outcome (GBS disability scale‡)
Presented case (Taiwan)	1/18,801 (ChAdOx1 nCoV-19)	41	M	ChAdOx1 (1st dose)	7	Paresthesia, facial diplegia (No)	Yes (159.8)	Bilateral facial palsy with paresthesia	Equivocal	NA	NA	IVIG (0.4g/kg/day for 5 days)	No	Survived (1)
Theuriet J, et al (France) (*1*)	NA	72	M	ChAdOx1 (1st dose)	21	Myalgia, paresthesia, dysarthria, ascending paralysis, facial diplegia (No)	Yes (62.0)	Classic GBS	AIDP	1	Anti-GM3 IgM positive	IVIG (0.4g/kg/day for 5 days)	No	Survived (NA)
Hasan T, et al (UK) (*2*)	NA	62	F	ChAdOx1 (1st dose)	8	Paresthesia, ascending paralysis (No)	Yes (90.0)	Classic GBS	AIDP	1	NA	IVIG (0.4g/kg/day for 5 days)	Yes	Survived (5)
Allen CM, et al (UK) (*3*)	4/712,050 (ChAdOx1 nCoV-19)	54	M	ChAdOx1 (1st dose)	12	Paresthesia, facial diplegia (No)	Yes (162.6)	Bilateral facial palsy with paresthesia	Equivocal	NA	Negative	Prednisolone (60mg for 5 days)	No	Survived (NA)
		20	M	ChAdOx1 (1st dose)	22	Paresthesia, facial diplegia (No)	Yes (123.2)	Bilateral facial palsy with paresthesia	Equivocal	NA	Negative	Prednisolone (60mg for 5 days)	No	Survived (NA)
		57	M	ChAdOx1 (1st dose)	15	Back pain, paresthesia, dysarthria, facial diplegia (No)	Yes (247.1)	Bilateral facial palsy with paresthesia	Not done	NA	Negative	IVIG	No	Survived (NA)

Author name (country)	GBS rate	Age, y	Sex	Vaccine	Vaccination to symptom onset, d	Initial symptoms (dysautonomia)	Albuminocytologic dissociation (CSF protein, mg/dl)	Clinical classification	Electro-diagnosis	Brighton criteria†	Antiganglioside antibody	Treatment (drug dose)	Mechanical ventilation	Outcome (GBS disability scale‡)
		55	M	ChAdOx1 (1st dose)	22	Paresthesia, facial diplegia no	Yes (89.0)	Bilateral facial palsy with paresthesia	Not done	NA	Negative	No treatment	No	Survived (NA)
Maramattom BV, et al (India) (*4*)	7/1,200,000 (ChAdOx1 nCoV-19)	43	F	ChAdOx1 (1st dose)	10	Back pain, quadriparesis, facial diplegia (No)	Yes (85.0)	Classic GBS	AIDP	1	NA	IVIG	Yes	Survived (0)
		67	F	ChAdOx1 (1st dose)	14	Paresthesia, quadriparesis, facial diplegia (No)	Yes (345.0)	Classic GBS	AMSAN	1	Negative	IVIG (NA) Plasmapheresis	Yes	Survived (4)
		53	F	ChAdOx1 (1st dose)	12	Paresthesia, quadriparesis, facial diplegia (No)	Yes (120.0)	Classic GBS	AIDP	1	Negative	IVIG (NA)	Yes	Survived (4)
		68	F	ChAdOx1 (1st dose)	14	Paresthesia, quadriparesis, dysphagia, facial diplegia (No)	Yes (75.0)	Classic GBS	AIDP	2	Negative	IVIG (NA)	Yes	Survived (4)
		70	M	ChAdOx1 (1st dose)	11	Paresthesia, quadriparesis, facial diplegia (No)	NA	Classic GBS	AIDP	2	NA	IVIG (NA)	Yes	Survived (4)
		69	F	ChAdOx1 (1st dose)	12	Paresthesia, quadriparesis, facial diplegia (No)	NA	Classic GBS	AIDP	2	NA	IVIG (NA) Plasmapheresis	No	Survived (4)
		69	F	ChAdOx1 (1st dose)	13	Paresthesia, quadriparesis, facial diplegia (No)	Yes (83.0)	Classic GBS	AIDP	1	NA	IVIG (NA)	Yes	Survived (5)
Ogbebor O, et al (USA) (*5*)	NA	86	F	BNT162b2 (1st dose)	1	Paraparesis (No)	Yes (162.0)	Paraparetic	Not done	2	NA	IVIG (NA)	No	Survived (3)
Patel SU, et al (UK) (*6*)	NA	37	M	ChAdOx1 (1st dose)	14	Back pain, paresthesia, ascending paralysis (No)	Yes (177.0)	Classic GBS	Not done	2	NA	IVIG (2g/kg/day for 5 days)	No	Survived (NA)
Márquez Loza AM, et al (USA) (*7*)	NA	60	F	Ad26.COV2.S (1st dose)	10	Back pain, paraparesis, facial diplegia (No)	Yes (140.0)	GBS / MFS overlap variants	AIDP	1	Negative	IVIG (2g/kg/day for 2 days)	No	Survived (NA)

Author name (country)	GBS rate	Age, y	Sex	Vaccine	Vaccination to symptom onset, d	Initial symptoms (dysautonomia)	Albuminocytologic dissociation (CSF protein, mg/dl)	Clinical classification	Electro-diagnosis	Brighton criteria†	Antiganglioside antibody	Treatment (drug dose)	Mechanical ventilation	Outcome (GBS disability scale‡)
Waheed S, et al (USA) (*8*)	NA	82	F	BNT162b2 (1st dose)	14	Myalgia, paresthesia, paraparesis BP fluctuation	Yes (88.0)	Paraparetic	Not done	2	NA	IVIG (NA)	No	Survived (NA)
Razok A, et al (Qatar) (*9*)	NA	73	M	BNT162b2 (2nd dose)	16	Paraparesis (No)	Yes (80.0)	Paraparetic	AIDP	1	NA	IVIG (0.4g/kg/day for 5 days)	No	Survived (0)
Nasuelli NA, et al (Italy) (*10*)	NA	59	M	ChAdOx1 (1st dose)	10	Paresthesia, facial diplegia (No)	Yes (140)	Bilateral facial palsy with paresthesia	AIDP	NA	Negative	IVIG (0.4g/kg/day for 5 days)	No	Survived (NA)
Bonifacio GB, et al (UK) (*11*)	NA	66	M	ChAdOx1 (1st dose)	7	Back pain, paresthesia, facial diplegia (No)	Yes (199)	Bilateral facial palsy with paresthesia	AIDP	NA	Negative	IVIG (NA)	No	Survived (1)
		43	M	ChAdOx1 (1st dose)	11	Myalgia, paresthesia, facial diplegia Urinary retention	Yes (281)	Bilateral facial palsy with paresthesia	AIDP	NA	Negative	IVIG (NA)	No	Survived (2)
		51	M	ChAdOx1 (1st dose)	7	Myalgia, paresthesia, facial diplegia (No)	Yes (514)	Bilateral facial palsy with paresthesia	AIDP	NA	Anti-GM3 positive, anti-GM4 borderline	No treatment	No	Survived (2)
		71	F	ChAdOx1 (1st dose)	12	Back pain, Paresthesia, facial diplegia (No)	Yes (96)	Bilateral facial palsy with paresthesia	AIDP	NA	Negative	No treatment	No	Survived (2)
		53	M	ChAdOx1 (1st dose)	8	Paresthesia, facial diplegia (No)	Yes (122)	Bilateral facial palsy with paresthesia	Not done	NA	Negative	No treatment	No	Survived (1)
McKean N, et al (Malta) (*12*)	NA	48	M	ChAdOx1 (1st dose)	10	Back pain, facial diplegia, paresthesia, quadriparesis (No)	Yes (126.4)	Classic GBS	AIDP	1	Negative	IVIG (2g/kg/day for 5 days)	No	Survived (1)
James J, et al (India) (*13*)	NA	60	M	ChAdOx1 (1st dose)	11	Quadriparesis, paresthesia, facial diplegia (No)	Yes (149)	Classic GBS	AMSAN	1	NA	IVIG (2g/kg/day for 5 days)	No	Survived (3)

Author name (country)	GBS rate	Age, y	Sex	Vaccine	Vaccination to symptom onset, d	Initial symptoms (dysautonomia)	Albuminocytologic dissociation (CSF protein, mg/dl)	Clinical classification	Electro-diagnosis	Brighton criteria†	Antiganglioside antibody	Treatment (drug dose)	Mechanical ventilation	Outcome (GBS disability scale‡)
		66	M	ChAdOx1 (1st dose)	12	Ascending paralysis, paresthesia Urinary retention, BP fluctuation	Yes (84)	Classic GBS	AIDP	1	NA	IVIG (2g/kg/day for 5 days), Methylprednisolone (1000 mg for 3 days)	No	Survived (3)
		54	F	ChAdOx1 (1st dose)	13	Quadriparesis, paresthesia, dysphagia (No)	NA	Classic GBS	AIDP	2	Negative	IVIG (2g/kg/day for 5 days), Methylprednisolone (1000 mg for 3 days)	No	Survived (2)
García-Grimshaw M, et al (Mexico) (*14*)	7/3,890,250 (BNT162b2)	33	M	BNT162b2 (1st dose)	28	Facial diplegia (No)	Yes (67.1)	Bilateral facial palsy with paresthesia	AIDP	NA	NA	IVIG (NA)	No	Survived (1)
		25	M	BNT162b2 (1st dose)	12	Paresthesia, quadriparesis (No)	Yes (64)	Classic GBS	AIDP	1	NA	IVIG (NA)	No	Survived (3)
		53	F	BNT162b2 (1st dose)	6	Quadriparesis (No)	No (15)	Classic GBS	AMAN	2	NA	IVIG (NA)	Yes	Survived (5)
		72	M	BNT162b2 (1st dose)	4	Quadriparesis (No)	NA	Classic GBS	AMAN	2	NA	IVIG (NA)	No	Survived (4)
		31	M	BNT162b2 (1st dose)	11	Quadriparesis (No)	NA	Classic GBS	AIDP	2	NA	IVIG (NA)	No	Survived (3)
		67	F	BNT162b2 (1st dose)	4	Quadriparesis (No)	No (30)	Classic GBS	AMAN	2	NA	IVIG (NA)	Yes	Expired (6)
		81	F	BNT162b2 (1st dose)	3	Quadriparesis (No)	Yes (414)	Classic GBS	AIDP	1	NA	IVIG (NA)	No	Survived (4)
Trimboli M, et al (Italy) (*15*)	NA	25	F	BNT162b2 (2nd dose)	5	Paraparesis, paresthesia (No)	No (NA)	Paraparetic	AIDP	2	NA	IVIG (0.4g/kg/day for 5 days)	No	Survived (2)
Tutar NK, et al (Turkey) (*16*)	NA	76	M	CoronaVac (2nd dose)	5	Myalgia, ascending paralysis (No)	No (NA)	Classic GBS	AMSAN	2	Negative	IVIG (2g/kg/day for 5 days)	No	Survived (2)

Author name (country)	GBS rate	Age, y	Sex	Vaccine	Vaccination to symptom onset, d	Initial symptoms (dysautonomia)	Albuminocytologic dissociation (CSF protein, mg/dl)	Clinical classification	Electro-diagnosis	Brighton criteria†	Antiganglioside antibody	Treatment (drug dose)	Mechanical ventilation	Outcome (GBS disability scale‡)
Scendoni R, et al (Italy) (*17*)	NA	82	F	BNT162b2 (2nd dose)	15	Ascending paralysis, paresthesia (No)	Yes (570)	Classic GBS	AMSAN	1	Anti-sulfatide IgG and IgM positive, anti-GM2 IgM positive, anti-GM4 IgM positive	IVIG (0.4g/kg/day for 5 days)	No	Survived (4)

*AIDP, acute inflammatory demyelinating polyneuropathy; AMAN, acute motor axonal neuropathy; AMSAN, acute motor and sensory axonal neuropathy; BP, blood pressures; CSF, cerebral spinal fluid; GBS, Guillain-Barré Syndrome; IVIG, Intravenous immunoglobulin; MFS, Miller-Fisher Syndrome; NA, not available.

†The Brighton Criteria is a diagnostic tool for GBS, ranging from level 1 (highest level) to level 4 (lowest level) of diagnostic certainty by assessing the patient's clinical presentation, cerebrospinal fluid data and nerve conduction velocity findings.

‡GBS Disability Scale grades the patient's functional disability from 0 (healthy) to 6 (dead).

References

1. Theuriet J, Richard C, Becker J, Pegat A, Bernard E, Vukusic S. Guillain158 Barré syndrome following first injection of ChAdOx1 nCoV-19 vaccine: 159 first report. Rev Neurol (Paris). 2021 Jun 24 [Epub ahead of print].

2. Hasan T, Khan M, Khan F, Hamza G. Case of Guillain-Barré syndrome following COVID-19 vaccine. BMJ Case Rep. 2021;14:e243629. PubMed https://doi.org/10.1136/bcr-2021-243629

3. Allen CM, Ramsamy S, Tarr AW, Tighe PJ, Irving WL, Tanasescu R, et al. Guillain-Barré syndrome variant occurring after SARS-CoV-2 vaccination. Ann Neurol. 2021;90:315–8. PubMed https://doi.org/10.1002/ana.26144

4. Maramattom BV, Krishnan P, Paul R, Padmanabhan S, Cherukudal Vishnu Nampoothiri S, Syed AA, et al. Guillain-Barré Syndrome following ChAdOx1-S/nCoV-19 vaccine. Ann Neurol. 2021;90:312–4. PubMed https://doi.org/10.1002/ana.26143

5. Ogbebor O, Seth H, Min Z, Bhanot N. Guillain-Barré syndrome following the first dose of SARS-CoV-2 vaccine: a temporal occurrence, not a causal association. IDCases. 2021;24:e01143. PubMed https://doi.org/10.1016/j.idcr.2021.e01143

6. Patel SU, Khurram R, Lakhani A, Quirk B. Guillain-Barre syndrome following the first dose of the chimpanzee adenovirus-vectored COVID-19 vaccine, ChAdOx1. BMJ Case Rep. 2021;14:e242956. PubMed https://doi.org/10.1136/bcr-2021-242956

7. Márquez Loza AM, Holroyd KB, Johnson SA, Pilgrim DM, Amato AA. Guillain-Barré syndrome in the placebo and active arms of a COVID-19 vaccine clinical trial: temporal associations do not imply causality. Neurology. 2021 Apr 6 [Epub ahead of print].

8. Waheed S, Bayas A, Hindi F, Rizvi Z, Espinosa PS. Neurological complications of COVID-19: Guillain-Barre syndrome following Pfizer COVID-19 vaccine. Cureus. 2021;13:e13426. PubMed

9. Razok A, Shams A, Almeer A, Zahid M. Post-COVID-19 vaccine Guillain-Barré syndrome; first reported case from Qatar. Ann Med Surg (Lond). 2021;67:102540. PubMed https://doi.org/10.1016/j.amsu.2021.102540

10. Nasuelli NA, De Marchi F, Cecchin M, De Paoli I, Onorato S, Pettinaroli R, et al. A case of acute demyelinating polyradiculoneuropathy with bilateral facial palsy after ChAdOx1 nCoV-19 vaccine. Neurol Sci. 2021 Jul 17 [Epub ahead of print]. PubMed

11. Bonifacio GB, Patel D, Cook S, Purcaru E, Couzins M, Domjan J, et al. Bilateral facial weakness with paraesthesia variant of Guillain-Barré syndrome following Vaxzevria COVID-19 vaccine. J Neurol Neurosurg Psychiatry. 2021 Jul 14 [Epub ahead of print]. PubMed https://doi.org/10.1136/jnnp-2021-327027

12. McKean N, Chircop C. Guillain-Barré syndrome after COVID-19 vaccination. BMJ Case Rep. 2021;14:e244125. PubMed https://doi.org/10.1136/bcr-2021-244125

13. James J, Jose J, Gafoor VA, Smita B, Balaram N. Guillain-Barré syndrome following ChAdOx1 nCoV-19 COVID-19 vaccination: a case series. Neurol Clin Neurosci. 2021;9:402–5. PubMed https://doi.org/10.1111/ncn3.12537

14. García-Grimshaw M, Michel-Chávez A, Vera-Zertuche JM, Galnares-Olalde JA, Hernández-Vanegas LE, Figueroa-Cucurachi M, et al. Guillain-Barré syndrome is infrequent among recipients of the BNT162b2 mRNA COVID-19 vaccine. Clin Immunol. 2021;230:108818. PubMed https://doi.org/10.1016/j.clim.2021.108818

15. Trimboli M, Zoleo P, Arabia G, Gambardella A. Guillain-Barré syndrome following BNT162b2 COVID-19 vaccine. Neurol Sci. 2021. PubMed https://doi.org/10.1007/s10072-021-05523-5

16. Tutar NK, Eyigürbüz T, Yildirim Z, Kale N. A variant of Guillain-Barre syndrome after SARS-CoV-2 vaccination: AMSAN. Ideggyogy Sz. 2021;74:286–8. PubMed https://doi.org/10.18071/isz.74.0286

17. Scendoni R, Petrelli C, Scaloni G, Logullo FO. Electromyoneurography and laboratory findings in a case of Guillain-Barré syndrome after second dose of Pfizer COVID-19 vaccine. Hum Vaccin Immunother. 2021 Aug 4 [Epub ahead of print]. PubMed https://doi.org/10.1080/21645515.2021.1954826
